# Basal hyperinsulinemia beyond a threshold predicts major adverse cardiac events at 1 year after coronary angiogram in type 2 diabetes mellitus: a retrospective cohort study

**DOI:** 10.1186/s13098-017-0237-x

**Published:** 2017-05-19

**Authors:** Mukund Srinivasan, Padmanabh Kamath, Narayan Bhat, Narasimha Pai, Rajesh Bhat, Tejas Shah, Poornima Manjrekar, Chakrapani Mahabala

**Affiliations:** 1 Department of Internal Medicine, Kasturba Medical College, Manipal University, Mangalore, Karnataka 575001 India; 2 Department of Cardiology, Kasturba Medical College, Manipal University, Mangalore, Karnataka India; 3 Department of Biochemistry, Kasturba Medical College, Manipal University, Mangalore, Karnataka India

**Keywords:** Coronary artery disease, Hyperinsulinemia, Major adverse cardiac events, Type 2 diabetes mellitus

## Abstract

**Background:**

There is a substantial reduction in cardiovascular related morbidity and mortality in the general population attributed to improved treatment of cardiac risk factors and disease, the same magnitude of benefit has not been observed in those with diabetes mellitus. The aim of the present study was to evaluate factors associated with the cardiac outcome at 1 year after coronary angiogram in patients with type 2 diabetes mellitus and to compare the outcomes with nondiabetics.

**Methods:**

A retrospective cohort study was carried out in subjects who underwent coronary angiogram for an evaluation of CAD, with follow-up data available for period of 12 months. The data consisted of 208 type 2 diabetic and 75 non-diabetic patients. Clinical, anthropometric and other biochemical risk factors of the study participants were recorded. Univariate and multivariate cox proportional hazard regression analyses were performed to evaluate the relation between the cardiovascular risk factors and major adverse cardiac events (MACE).

**Results:**

At 1 year, MACE was observed in 50 (24.04%) type 2 diabetic subjects, which included non-fatal myocardial infarction 24 (11.54%), target vessel revascularization 15 (7.21%) and death 11 (5.29%). The area under the curve for insulin in predicting MACE was found to be 0.81 (95% CI 0.73–0.88) with sensitivity and specificity of 88% (95% CI 0.71–0.96) and 74% (95% CI 0.65–0.81) respectively. After adjustment for potential confounders hyperinsulinemia (>20 µIU/ml) was significantly associated with MACE [adjusted hazard ratio (HR): 3.03, 95% CI 1.41–6.54, p = 0.005]. Interestingly, the MACE rate in type 2 diabetics with insulin levels <20 µIU/ml (10.2%) and non-diabetics (12%) (p = 0.676) appears to be same.

**Conclusions:**

In addition to severity of the CAD at the baseline, basal hyperinsulinemia beyond a threshold strongly predicts adverse cardiac events at 1 year in type 2 diabetes mellitus. Those below the threshold, appears to be having a risk equivalent to non-diabetics.

## Background

The risk for major adverse cardiac events (MACE) are two to fourfold higher in type 2 diabetes mellitus even after adjusting for the known conventional cardiovascular risk factors. Several long term trials have shown that diabetics have high rate of adverse cardiac events even after undergoing successful revascularization procedures [1–4].

However, factors driving this complication are not fully understood. Since individuals with type 2 diabetes mellitus have been shown to display more diffuse and calcified disease than non-diabetics, it is assumed that a severe baseline disease is the cause for this excess adverse cardiac events in type 2 diabetic patients. But the role of other clinical and biochemical risk factors on MACE in this patient population is not fully elucidated.

While a few epidemiological studies have shown that hyperinsulinemia has been associated with new adverse cardiac events in non-diabetics, and in general population [5–8], the same has not been demonstrated in type 2 diabetic patients after undergoing coronary angiogram. Despite the results of these epidemiological studies establish hyperinsulinemia as an independent predictor of cardiovascular outcomes, it could not provide the biological explanation between high insulin level and CVD risk. Of importance several molecular mechanism have been proposed which explains the effects of elevated insulin levels on the vasculature leading to an increased atherogenesis [9]. Studies in both men and women have shown that high fasting insulin levels are directly associated with carotid intima thickness and arterial wall stiffness even after adjusting for hypertension, dyslipidemia and obesity. Since in type 2 diabetics preexisting hyperinsulinemia is observed, it appears that these are the subjects who might be at higher risk for acute cardiovascular events [10–13].

Considering type 2 diabetes mellitus as a heterogeneous disease, it appears that not all people with type 2 diabetes mellitus are susceptible for adverse cardiac events [14], only a particular subset of individuals appears to be at risk for developing MACE, and the rate of MACE might also differ among diabetic population.

Since hyperinsulinemia/insulin resistance is known to be associated with the cardiac events, we hypothesize that, in addition to severity of the disease, there might be a threshold value for basal insulin/insulin resistance which is strongly associated with future adverse cardiac events and might play a role in prediction of clinical events in individuals with type 2 diabetes after undergoing coronary angiogram. Thus it might further increase the possibility of identifying high risk group and enable us to manage them aggressively to prevent any future adverse cardiac events.

## Methods

### Study participants and enrollment

The data for this retrospective cohort study consisted of 283 subjects who previously underwent coronary angiogram for an evaluation of clinically suspected coronary artery disease at a tertiary care hospital, from February 2013 to January 2015.

Among 283 subjects enrolled, 208 were found have type 2 diabetes mellitus and remaining 75 were non-diabetics. Type 2 diabetes mellitus was defined in accordance to the diagnostic criteria recommended by American Diabetes Association [15].

From the cohort of 208 type 2 diabetic patients, 128 patients underwent coronary angioplasty, 27 patients were subjected to coronary artery bypass graft (CABG) and remaining 53 were managed conservatively. Among 75 non-diabetic subjects, 56 underwent angioplasty, 5 were referred for CABG and 14 were considered for conservative management. The choice of different treatment modality was based on patient’s or treating cardiologist’s preference. In all cases of coronary angioplasty, patients were either deployed with Siroliums and Everolimus drug eluting stents (DES) or in some cases bio absorbable stents, none of the patients were subjected to bare metal stenting. The choice of specific type of DES was at the discretion of the operator.

### Inclusion and exclusion criteria

The study included all the participants whose baseline and follow-up records on were available. Patients on steroids, chronic kidney diseases, valvular heart diseases and on insulin were excluded. Ethical clearance to conduct the present study was obtained from Kasturba Medical College, Mangalore, Manipal University Ethics Committee. An informed consent form was obtained from study participants who were included in the study.

The data on clinical findings, anthropometric measurements and biochemical parameters such as fasting glycemia, fasting insulin, fasting lipid profile, glycated haemoglobin and urine microalbumin were noted. The baseline data was collected from study participants who were recruited for an earlier cross-sectional study [16]. The assessment and calculation of severity of CAD was determined by SYNTAX score, a web-based algorithm consisting of sequential and interactive self-guided questions (www.syntaxscore.com) [17].

### Follow-up at 1 year

The data of 208 type 2 diabetic patients who had undergone coronary angiogram for an evaluation of suspected CAD and who completed follow-up for a minimum period of 1 year was noted. 75 non-diabetic subjects full filling the inclusion criteria and who completed 1 year follow-up was also documented in order to compare their MACE rate with diabetic counterpart.

The primary outcome of the study was MACE at 1 year. MACE was defined as cardiac death, nonfatal myocardial infarction, or target lesion revascularization [18]. The myocardial infarction was defined as an increase in troponin or creatine kinase-MB >3 times [7–25 U/l], the upper normal limit, and subsequently myocardial infarction was identified by any elevation of troponin or creatine kinase-MB above the upper normal limit. The target lesion revascularization was defined as any repeat intervention (by coronary artery bypass graft or PCI) performed to treat a stenosis inside the implanted stent or within the 5-mm segments adjacent to the stent, including the ostium of the left anterior descending artery and/or left circumflex artery. The primary endpoint of the study was checked for accuracy, consistency, and completeness of follow-up by cardiologists who were blind to the patient’s baseline characters. All subjects received dual anti-platelet therapy (DAPT) [Clopidogrel 75 and Aspirin 75] for a minimum of 1 year.

### Statistical analysis

Data are presented as mean ± SD. Independent sample t test was performed to find out the mean differences between those who had adverse cardiac events and who did not have any cardiovascular events. The categorical variables were analysed by Chi square test. The normality assumption for continuous variables was evaluated by the Kolmogorov–Smirnov test. Receiver operating curve (ROC) was plotted to find out the optimal cut-off value for insulin level. Cox proportional hazard model was used to assess risk factors for adverse events. First, the univariate cox proportional was used to identify potential predictors of adverse cardiac events at 1 year. Then the variables with p value <0.20 in the univariate analysis, were included in the multivariate cox proportional hazard model to identify the potential predictors of adverse events at 1 year. The time to events were summarized and displayed using cumulative incidence curve by Kaplan–Meier survival analysis method. A p value <0.05 was considered to be statistically significant. Analysis was done using Statistical Package for Social Sciences (SPSS Version 15, Chicago IL).

## Results

Table 1 shows clinical characteristics of type 2 diabetic subjects with and without MACE. The mean follow-up of the study was 10.48 ± 3.18 months. At 1 year 50 (24.04%) type 2 diabetic subjects developed a new cardiovascular event. Majority among them had non-fatal myocardial infarction 24 (11.54%), followed by target vessel revascularization 15 (7.21%) and death from cardiac origin 11 (5.29%). There was a significant difference in mean fasting blood insulin (p < 0.01), syntax score (p < 0.01), duration of diabetes (p = 0.007), fasting blood glucose (p = 0.001), body mass index (p = 0.023) and left ventricular ejection fraction (p = 0.015) in those who suffered MACE when compared who did not have MACE (Table 1).

**Table 1:** Clinical characteristics of type 2 diabetic study participants with and without MACE

Variables	With MACE (n = 50)	Without MACE (n = 158)	p value
Duration of diabetes (years)	9.91 ± 6.87	6.92 ± 5.72	0.007
Fasting blood sugar (mg/dl)	208.82 ± 56.04	177.54 ± 55.94	0.001
Fasting insulin (IU/µl)	25.09 ± 7.2	18.52 ± 6.08	<0.001
Syntax score	24.14 ± 10.42	11.46 ± 8.97	<0.001
TC/HDL	4.19 ± 1.40	4.25 ± 1.55	0.803
LDL-C (mg/dl)	106.41 ± 40.51	100.69 ± 42.47	0.392
HbA1C	9.07 ± 1.54	8.95 ± 2.03	0.722
Microalbumin (mg/l)	58.55 ± 26.27	34.25 ± 18.29	0.266
Body mass index (kg/m^2^)	22.55 ± 3.03	23.66 ± 2.68	0.023
Waist circumference (cm)	87.92 ± 10.22	90.02 ± 7.83	0.186
Ejection fraction	46.58 ± 10.93	50.79 ± 8.66	0.015
Presence of hypertension (%)	21 (42%)	77 (48.7%)	0.406
Smoking (%)	8 (16%)	23 (14.6%)	0.803
Females (%)	15 (30%)	51 (32.3%)	–
Males (%)	35 (70%)	107 (67.7%)	–

*TC/HDL* total cholesterol/high density lipoprotein ratio, *LDL* low density lipoprotein, *HbA1c* hemoglobin A1C

**Fig. 1:**
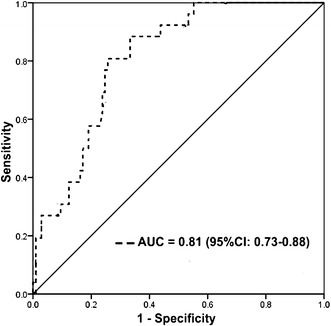
Receiver operating characteristic curves showing the performance of insulin in predicting major adverse cardiovascular events in type 2 diabetes mellitus

An area under the ROC curve for insulin predicting MACE was found to be statistically significant [AUC = 0.81 (95% CI 0.73–0.88)] (Fig. 1). A value of insulin >20 µIU/ml had sensitivity and specificity of 88% (95% CI 0.71–0.96) and 74% (95% CI 0.65–0.81) for MACE respectively.

**Fig. 2:**
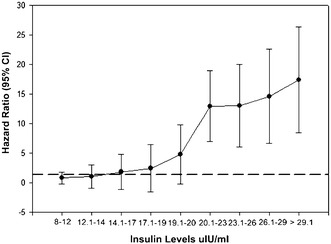
Hazard ratio for MACE by insulin deciles in type 2 diabetes mellitus

Adjusted hazard ratio for predicting MACE by insulin deciles is shown in Fig. 2. Insulin values in the lowest deciles (8–12) were not associated with a heightened risk for adverse cardiac events. A clear risk of adverse cardiac events increased beyond an insulin level of 20 µIU/ml (Fig. 2). Further, based on this threshold level, 119 (57%) type 2 diabetic subjects were found to have insulin levels <20 µIU/ml and remaining 89 (43%) had insulin levels >20 µIU/ml respectively.

Table 2 shows the clinical characteristics of non-diabetic study population. Among 75 non diabetic subjects, 9 (12%) of them developed new cardiovascular events at 1 year, 4 (5.33%) had non-fatal myocardial infarction, 3 (4%) had target vessel revascularization and 2 (2.67%) death from cardiac origin respectively.

**Table 2:** Clinical characteristics of non-diabetic study participants

Variables	Mean ± standard deviation (N = 75)
Age (years)	56.07 ± 5.78
Fasting blood sugar (mg/dl)	111.40 ± 37.45
Syntax score	12.43 ± 7.04
TC/HDL	4.41 ± 1.37
LDL-C (mg/dl)	113.92 ± 37.45
HbA1C	5.79 ± 0.79
Microalbumin (mg/l)	42.44 ± 10.74
Body mass index (kg/m^2^)	23.26 ± 3.66
Waist circumference (cm)	83.78 ± 4.98
Ejection fraction	47.58 ± 9.93
Presence of hypertension (%)	25 (33.33%)
Smoking (%)	33 (44%)
Females (%)	13 (17.33%)
Males (%)	62 (82.67%)

*TC/HDL* total cholesterol/high density lipoprotein ratio, *LDL* low density lipoprotein, *HbA1c* hemoglobin A1C

**Fig. 3:**
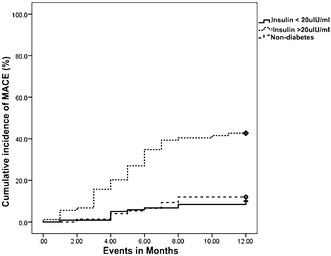
Cumulative incidence of MACE at 1 year based on insulin levels in type 2 diabetes mellitus and non-diabetes

Type 2 diabetic subjects experienced higher MACE rate (24%) than non-diabetes (12%) (p = 0.028). However, subgroup analysis based on insulin levels in type 2 diabetes mellitus showed, patients with insulin levels beyond >20 µIU/ml had a higher MACE rate 42.4% (38) compared to those with insulin levels <20 µIU/ml [10.2% (12)] (p < 0.001). The MACE rate between those with insulin levels <20 µIU/ml (10.2%) and non-diabetics (12%) were found to be similar (p = 0.676). The cumulative incidence of MACE at 1 year for non-diabetics, insulin levels <20 and >20 µIU/ml is shown in Fig. 3.

Table 3 shows the univariate cox-proportional hazard model for prediction of MACE at 1 year in type 2 diabetes mellitus. Those variables with p value <0.20 were included for multivariate cox proportional hazard model.

**Table 3:** Univariate Cox regression analysis for the relationship between adverse cardiovascular events and other conventional risk factors in type 2 diabetes mellitus

Variables	B	Hazard ratio	p value	95% CI
Syntax score >22	1.60	4.95	<0.001	2.82–8.68
Insulin >20 IU/µl	1.64	5.18	<0.001	2.70–9.92
Microalbumin >20 mg/l	0.650	1.91	0.029	1.06–3.44
Duration of diabetes >5 years	0.850	2.33	0.007	1.26–4.3
Mode of treatment (PTCA/CABG/medical)	0.792	2.20	0.005	1.26–3.86
TC/HDL >5	−0.424	0.65	0.249	0.32–1.34
LDL >100 mg/dl	0.457	1.57	0.111	0.90–2.76
Body mass index >23 kg/m^2^	−0.496	0.61	0.086	0.34–1.07
Waist circumference (cm)	−0.229	0.79	0.553	0.37–1.69
Hypertension	−0.225	0.80	0.432	0.45–1.4
Smoking	0.079	1.08	0.837	0.50–2.36
Gender	0.049	1.05	0.874	0.57–1.92
Age	0.032	1.03	0.243	0.98–1.08

*TC/HDL* total cholesterol/high density lipoprotein ratio, *LDL* low density lipoprotein

After adjusting for potential confounders in multivariate cox proportional hazard model, the fasting insulin >20 µIU/ml [adjusted hazard ratio (HR): 3.03, 95% CI 1.41–6.54, p = 0.005] and severity of CAD [adjusted hazard ratio (HR): 2.32, 95% CI 1.07–5.02, p = 0.032] were significantly associated with MACE at 1 year in type 2 diabetes mellitus (Table 4). The other conventional risk factors of CAD were not statistically significant (Table 4).

**Table 4:** Multivariate Cox regression analysis showing the independent predictors of adverse cardiovascular events in type 2 diabetes mellitus: result of a 1 year follow-up

Variables	B	Hazard ratio	p value	95% CI
Insulin >20 IU/µl	1.11	3.03	0.005	1.41–6.54
Syntax score >22	0.84	2.32	0.032	1.07–5.02
Microalbumin >20 mg/l	0.43	1.54	0.153	0.85–2.81
Duration of diabetes >5 years	0.28	1.32	0.415	0.67–2.62
Mode of treatment (PTCA)	0.02	1.02	0.970	0.42–2.48
Mode of treatment (medical)	0.28	1.32	0.508	0.58–3.04
LDL >100 mg/dl	0.14	1.15	0.641	0.64–2.08
Body mass index >23 kg/m^2^	−0.49	0.61	0.109	0.34–1.11

*LDL* low density lipoprotein

## Discussion

A substantial reduction in cardiovascular mortality and morbidity has been achieved in general population which is attributed to an improvement in treatment for cardiovascular risk factors and disease. However, the same magnitude of benefit has not been demonstrated in type 2 diabetes mellitus [1, 2, 19], and factors that are responsible for these new cardiovascular events have not been fully elucidated in this population.

Our study reveals that, in addition to severity of the disease, a basal insulin level beyond a threshold of 20 µIU/ml is a significant predictor of new adverse cardiac events at 1 year in type 2 diabetic subjects, after adjusting for other potential risk factors.

Several longitudinal studies have shown that, hyperinsulinemia is associated with new cardiac events in general population [5–8]. In a prospective study, fasting immune reactive insulin levels beyond 20 µIU/ml was independently associated with an incidence of CAD in insulin treated diabetic cohort [20]. Subjects with high insulin levels were at five to sixfold risk for developing CAD [20]. Further, in a cross-sectional study it was observed that an elevated insulin level was independently associated with angiographically determined CAD [21]. The findings derived from our study are in concordance to an earlier studies, and shows that increased insulin is not only associated with incidence of CAD, but also predicts adverse cardiac events at 1 year after coronary angiogram in type 2 diabetes mellitus.

In addition, the present study has identified a basal insulin threshold level. Hyperinsulinemia is a common condition often associated with T2DM in which insulin levels exceed the normal range. However, until the insulin levels reach peak values, there does not appear to be a risk of developing severe vascular complications. In the present study, insulin levels above 20 µIU/mL were potential predictors of MACE and insulin levels below that threshold were associated with risk rates for developing new cardiac events that were comparable risk rates for patients without diabetes.

In Verona diabetes study, insulin resistance was independently predicted cardiovascular disease in type 2 diabetes mellitus [22]. Insulin resistance was measured at a mean time of 9.3 years after diagnosis of diabetes. In this present study basal insulin was measured at a mean duration of 8.6 years of type 2 diabetes mellitus which is in compliance with Verona diabetes study, and thus we were able to establish an association between hyperinsulinemia/insulin resistance with adverse cardiac events in these patient cohort.

Inspite of undergoing successful coronary interventions the chances for developing new adverse cardiac events still remain high in type 2 diabetes mellitus. It has been speculated that, it is the severity of the disease and type of coronary interventions that are mainly associated with an increased adverse cardiac outcomes in this population [19, 23].

The Medicine, Angioplasty or Surgery Study (MASS) on multivessel CAD showed that, different treatment strategies did not influence cardiac outcomes in type 2 diabetes mellitus during the first year follow-up [24]. A 5-year prospective study in type 2 diabetic patients with the single vessel coronary stenting, the risk of adverse event was higher in type 2 diabetes mellitus, which is irrespective of its severity of the disease [25]. Further the risk of repeat revascularization was high only in the first year after single lesion stenting, but they were at increased risk for other clinical events including cardiac death and non-fatal MI over next 4 years [25].

Further, in a subgroup analysis of FREEDOM trial, type 2 diabetes mellitus with multi vessel CAD, the rate of MACE was higher in insulin provisioning treatment group than in those who were restricted for insulin treatment, even after adjusting for clinical demographics, severity of the disease and revascularization treatment [26]. No significant difference was observed in the magnitude of PCI or CABG benefit in type 2 diabetic patients with insulin treatment group [26].

However, majority of these studies did not identify the sub-group who might be at high risk for developing future complications. It appears that these are the individuals who might be having an insulin levels greater than 20 µIU/ml and hence did not derive significant benefit even after successful revascularization procedures.

The complications encountered in type 2 diabetes mellitus seems to be associated with the threshold level of basal insulin. At normal physiological level, insulin upon binding to its receptor initiates the vascular actions with the help of two major pathways. The mitogenic, proliferative and proinflammatory signalling pathway which is mediated by mitogen-activated protein kinase (MAPK) and the metabolic (glucose action) signaling pathway mediated by phosphatidylinositol 3-kinase (PI3K) [27]. To simulate production of the powerful vasodilator nitric oxide (NO), insulin acts on an endothelium via the phosphatidylinositol (PI) 3-kinase/Akt/NO pathway which is one of the important vascular actions of insulin. One primary feature during insulin resistance state is that, it is characterized by inhibition of PI3K-dependent signalling pathway while other insulin-signalling pathways combining the Ras/MAPK-dependent pathways remains active [27]. The continuous activation of MAPK pathway results in an increased expression of adhesion molecule (VCAM-I), smooth muscle proliferation and endothelial dysfunction which plays a key role in development of diabetic macrovascular disease [27]. This pathophysiologic implication is based on the reason that, during insulin resistance state, the basal levels of insulin are increased in order to maintain the glucose homeostasis in the vasculature and elsewhere, this compensatory hyperinsulinemia results in inhibition of PI3K and activation MAPK-dependent pathways. However level at which the MAPK pathway gets activated is not clear. We speculate that the MAPK pathway might be activated strongly, beyond threshold level of 20 µIU/ml insulin, which leads to fibrotic and proliferative changes in the vasculature.

We found that, an overall MACE rate in subjects with type 2 diabetes mellitus (24.04%) was higher in comparison to non-diabetes (12%). This concurs well with previous long term trials such as SYNTAX, CARDia, BARI 2D and FREEDOM [1–4]. In a similar observation by Lourenço et al., the MACE rate in diabetic population following acute coronary syndrome (ACS) at 1 year was found to be 20.4%, where blood sugar levels >130.5 mg/dl was an independent predictor for MACE [28].

Interestingly in our study, the subgroup analysis based on threshold level for insulin levels showed that, MACE rate was significantly higher in those with insulin >20 µIU/ml [42.4% (38)] when compared to insulin <20 µIU/ml [10.2% (12)] (p < 0.001). The MACE rate in those with insulin <20 µIU/ml [10.2% (12)] and non-diabetics [12% (9)] (p = 0.676) were found to be almost similar. This finding highlights that the type 2 diabetes is not a homogenous population, not all patients with type 2 diabetes mellitus and CAD would be at risk for developing adverse cardiac events, only particular sub-group appears to be at an increased risk for MACE. These are the individuals who are needed to be followed-up meticulously.

Till now hyperinsulinemia was associated with occurrence of CAD, but findings from our study showed that it has a major implications in terms of cardiovascular outcomes. In clinical practice this threshold value is a practical tool. It helps to identify high risk groups and intensify management efforts.

## Limitations

The present study has a few limitations. This study is considerably limited by the small number of study patients, and that reduces the power of the statistical analyses.

## Conclusions

In this group of patients, insulin threshold of 20 µIU/ml was an adverse outcome predictor at 1 year in type 2 diabetes mellitus with coronary artery disease. Those below the threshold, appears to be behave as non-diabetics.
